# Virus-Vectored Ebola Vaccines

**Published:** 2017

**Authors:** I.V. Dolzhikova, E.A. Tokarskaya, A. S. Dzharullaeva, A. I. Tukhvatulin, D. V. Shcheblyakov, O.L. Voronina, S. I. Syromyatnikova, S. V. Borisevich, V. B. Pantyukhov, V. F. Babira, L. V. Kolobukhina, B. S. Naroditsky, D. Y. Logunov, A. L. Gintsburg

**Affiliations:** Federal Research Centre of Epidemiology and Microbiology named after Honorary Academician N. F. Gamaleya, Ministry of Health, Gamaleya Str. 18, Moscow, 123098, Russia; 48 Central Research Institute, Ministry of Defense, Oktjabr’skaja Str. 11, Sergiev Posad-6, Moscow oblast, 141306, Russia; No. 7 Main Military Clinical Hospital named after academician N. N. Burdenko, Ministry of Defense, Novaja Str. 4, Sergiev Posad-6, Moscow oblast, 141306, Russia; Infectious Clinical Hospital № 1, Moscow Healthcare Department, Volokolamskoe shosse, 63, Moscow, 125367, Russia

**Keywords:** Ebola vaccines, virus-vectored vaccines, recombinant viral vectors, Ebola virus

## Abstract

The Ebola virus disease (EVD) is one of the most dangerous infections affecting humans and animals. The first EVD outbreaks occurred in 1976 in Sudan and Zaire. Since then, more than 20 outbreaks have occurred; the largest of which (2014−2016) evolved into an epidemic in West Africa and claimed the lives of more than 11,000 people. Although vaccination is the most effective way to prevent epidemics, there was no licensed vaccine for EVD at the beginning of the latest outbreak. The development of the first vaccines for EVD started in 1980 and has come a long technological way, from inactivated to genetically engineered vaccines based on recombinant viral vectors. This review focuses on virus-vectored Ebola vaccines that have demonstrated the greatest efficacy in preclinical trials and are currently under different phases of clinical trial. Particular attention is paid to the mechanisms of immune response development, which are important for protection from EVD, and the key vaccine parameters necessary for inducing long-term protective immunity against EVD.

## EBOLA VIRUS DISEASE

The Ebola virus causes one of the most dangerous diseases affecting humans and primates. The Ebola virus disease (EVD) is characterized by a severe course, general intoxication, and a high mortality rate reaching 90% [1-3]. The genus Ebola virus (*Ebolavirus*) is a member of the Filoviridae family. Viral particles of all viruses from the Filoviridae family (order Mononegavirales) have a characteristic filament-like shape, and their genome is represented by a single-stranded RNA with negative polarity. There are three filovirus genera: *Ebolavirus*, *Marburgvirus*, and *Cuevavirus*. Of these, *Ebolaviruses *and *Marburgviruses *have marked pathogenicity to humans, and the Ebola virus (EBOV) is the most dangerous pathogen. To date, five Ebola virus species have been identified: *Bundibugyo ebolavirus *(BDBV), *Zaire ebolavirus *(ZEBOV), *Reston ebolavirus *(RESTV), *Sudan ebolavirus *(SUDV), and *Tai Forest ebolavirus *(TAFV); of these, ZEBOV, SUDV, and BDBV are the most dangerous for humans [4, 5].

EVD was first detected in Yambuku (Democratic Republic of the Congo, the northern part of Zaire) and in Nzara (Sudan) in 1976. In the same year, the EVD agent, Ebola virus (*Ebolavirus*), was first isolated from a patient who lived near the Ebola River [6, 7].

Since the time of pathogen isolation and to this day, more than 20 EVD outbreaks have occurred, the largest of which (2014–2016) turned into an epidemic (28,616 cases) and claimed the lives of more than 11,000 people [8]. By the time of this epidemic, neither preventive nor therapeutic agents for EVD were licensed in the world. At the same time, a specific heterologous (horse) immunoglobulin against Ebola fever was developed at the Virology Center of the Research Institute of the Russian Defense Ministry for urgent prophylaxis and treatment of high-risk groups; the immunoglobulin had 100% protective activity in experiments with monkeys [9]. Due to the high mortality rate in the last EVD epidemic and spread of the virus outside Africa, a WHO Committee was convened in early August 2014. The Committee concluded that the EVD outbreak was an extraordinary event of international importance, which significantly accelerated the development of preventive and therapeutic agents for EVD. After 2 years, several vaccines had been developed. They are currently under different phases of clinical trial, and two vaccines developed in Russia have been registered for medical use.

## EVD VACCINES: HISTORY AND DEVELOPMENT STRATEGIES

The most effective and economical way to protect against infectious diseases is vaccine prevention. However, there was no vaccine approved for use by the beginning of the last Ebola outbreak (2014–2016).

**Fig. 1:**
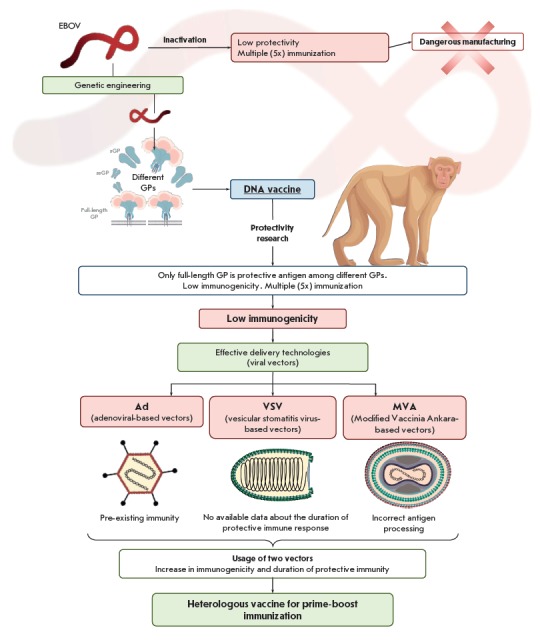
Ebola vaccine development strategies

The development of the first vaccines for Ebola fever began after the identification of the virus and was mainly focused on attempts to create an effective vaccine based on an inactivated Ebola virus (*Fig. 1*). In 1980, the first candidate vaccine on the basis of a heat- or formalin-inactivated Ebola virus was tested on guinea pigs and exhibited 100% protection [10]. But despite the high efficacy in guinea pigs, the vaccine did not provide the proper level of protection to primates from lethal infection [11]. Another disadvantage of this vaccine was the extremely dangerous production condition. All these facts prevented the introduction of the vaccine into clinical practice (*Fig. 1*).

It took more than 15 years to develop an effective and safe vaccine. This was associated with the fact that the expression features of the main protective antigen, Ebola virus GP, remained unclear for a long time. The breakthrough came in 1995, when an article by V.E. Volchkov et al. [12] was published. It was shown that RNA editing by viral polymerase resulted in several GP forms, of which only 20% were the full-length envelope antigen GP [12] (*Fig. 1*). The same study found that GP expressed in eukaryotic cells undergoes extensive glycosylation, which subsequently happens to be critical for the preservation of immunogenicity and antigen protection [12, 13].

Understanding the biosynthesis peculiarities of various GP forms led, first of all, to the generation of candidate DNA vaccines. The plasmid vectors that were used for constructing the vaccines contained the full-length glycoprotein GP gene or the nucleoprotein gene of Ebola virus. These vaccines showed a sufficiently high protection level in animal studies, with the efficacy of the DNA vaccine carrying the Ebola virus glycoprotein GP gene being higher than that of the vaccine carrying the Ebola virus nucleoprotein NP gene [14]. However, the use of these vaccines required multiple (5 times) administration of the drug to achieve a high level of protection [15], which was a critical limiting factor for their effective use during epidemic development.

The problem of multiple vaccination was resolved as the recombinant viral vector technology was developed (*Figure 1*). In contrast to DNA vaccines, these vectors provide a high and long-lasting level of target transgene expression, which enables the induction of protective immunity after one or two immunizations [16-18]. Experiments with direct comparison demonstrated much faster formation of the immune response to a recombinant viral vector- based candidate vaccine compared to a plasmid DNA-based vaccine [16]. It should be noted that immunization was associated not only with the humoral immune response, but also with a more pronounced cellular (CD8+ and CD4+) immune response, which later occurred to be the key aspect of protection against Ebola fever. Various studies have demonstrated that it is cellular immunity that plays a key role in the formation of protective immunity to the Ebola virus [19, 20]. Directed depletion of CD3+ (CD8+ and CD4+) cells in monkeys immunized against EVD caused a decrease in vaccinated protective immunity, which resulted in the death of all the animals from Ebola virus infection. If only CD8+ cells were depleted, the protective response also decreased in immunized monkeys: 80% of the animals died. At the same time, passive transfer of high-titer polyclonal antibodies to the Ebola virus from vaccinated monkeys to naive ones provided incomplete protection against lethal infection: 75% of the animals died despite the high titers of common IgGs and NtAbs in peripheral blood serum [20]. The importance of cellular immunity was indirectly confirmed by the fact that the peripheral blood of people who survived EVD contained an increased number of specific CD8+ cells compared to the blood of healthy people. At the same time, the number of specific CD4+ cells did not actually increase [21].

Summarizing the more than thirty-year history of studies aimed at developing an effective EVD vaccine, it may be concluded that the “ideal” vaccine for Ebola fever should induce cellular and humoral immune responses, be administered a minimum number of times, and induce prolonged protective immunity.

The use of recombinant viral vectors provides all the indicated conditions; in this regard, the vaccines developed on the basis of these conditions were supported by the WHO as a promising direction for the development of Ebola vaccines during the last EVD epidemic.

## VECTORED VACCINES AGAINST EVD UNDER CLINICAL TRIALS

The bulk of the developed vaccines for Ebola fever are based on the use of recombinant viral vectors expressing the protective antigen GP, a full-length Ebola virus surface glycoprotein.

Phase 3 clinical trials of a recombinant vesicular stomatitis virus (VSV)-based vaccine have now been completed. VSV-based vaccines encoding GP of the Zaire (1995 Kikwit) and Sudan Ebola virus have demonstrated efficacy in a series of preclinical trials in primates [22, 23]. The high immunogenicity of a VSV-based vaccine has been demonstrated in a series of clinical trials [24, 25]: The vaccine induced a high level of GP-specific antibodies (*Table*) associated with protection in primate studies. Clinical trials conducted in Europe and Africa have demonstrated that the use of a VSV-based vaccine at various doses leads to the induction of the humoral immune response, with the levels of GP-specific antibodies being similar. Phase 3 clinical trials in Guinea (using ring vaccination) demonstrated 100% efficacy of the vaccine [26, 27].

A recombinant human adenovirus serotype 5 (Ad5)-based vaccine encoding full-length 2014 ZEBOV GP passed phase 1 clinical trials in China [28, 29]. Administration of a high vaccine dose (1.6 × 10^11^ vp) induced a high level of GP-specific antibodies at a titer of 1 : 1,306 in 100% of volunteers 1 month after vaccination, and the T-cell response had a maximum on day 14 but decreased by day 28 of the study. Six months after vaccination, the GP antibody titer significantly decreased and amounted to 1 : 198 (*Table*). The volunteers were re-vaccinated 6 months after the primary vaccination. Four weeks after the re-vaccination, the vaccine induced a high level of GP-specific antibodies (titer of 1 : 11,825) in the blood serum of the volunteers. One year after the revaccination, the titer of GP-specific antibodies in the blood serum of the volunteers was 1 : 857. One of the main problems limiting the use of Ad5-based vectors is a wide prevalence of pre-existing immunity to Ad5 (the presence of Ad5 neutralizing antibodies) in the population. The presence of Ad5 antibodies before vaccination was shown to lead to the induction of a lower GP-specific humoral and T-cell response after vaccination [29, 30]. However, clinical trials in China demonstrated that the use of a high dose of an Ad5- based vaccine may reduce the negative effect of pre-existing immunity on the formation of a GP-specific immune response [29].

**Table:** Vaccines under different phases of clinical trials

Vaccine	Dose	Antigen origin	Immune response (titer of EBOV GP-specific IgGs)	Most common AE	Reference
Ad5	Low (2 × 10^9^ vp) High (2 × 10^10^ vp)	EBOV SUDV	GMT: Low: 85 (Day 28) High: 155 (Day 28)	Headache	[30]
Ad5	Low (4 × 10^10^ vp) High (1.6 × 10^11^ vp)	EBOV	GMT: Low: 682.7 (Day 28) High: 1,305.7 (Day 28)	Pain at the injection site	[28]
Ad5	Prime-boost (6 months) Low (4 × 10^10^ vp) High (1.6 × 10^11^ vp)	EBOV	GMT: Low: 682.7 (Day 28) 575.5 (6 months) 6,110 (Day 28 after boosting) 674.1 (12 months after boosting) High: 1,305.7 (Day 28) 197.9 (6 months) 11,825 (Day 28 after boosting) 856.8 (12 months after boosting)	Pain at the injection site	[29]
Ad26+MVA	Group 1 Priming: Ad26 (5 × 10^10^ vp) Boosting: MVA (108 TCID50) Group 2: Priming: MVA (108 TCID50) Boosting: Ad26 (5 × 10^10^ vp)	EBOV; SUDV; MARV; TAFV	GMC: Group 1: 7,553 (Day 21) Group 2: 18,474 (Day 21)	Pain at the injection site	[41]
ChAd3	Low (2 × 10^10^ vp) High (2 × 10^11^ vp)	EBOV; SUDV	GMT: Low: 331 (Day 28) High : 2,037 (Day 28)	Fever	[32]
ChAd3+MVA	Priming: ChAd3 Group 1 (1 × 10^10^ vp) Group 2 (2.5 × 10^10^ vp) Group 3 (5 × 10^10^ vp) Boosting: MVA 1.5 × 10^8^ PFU 3 × 10^8^ PFU	EBOV; SUDV; MARV; TAFV	GMT: Priming: 758 (6 months) Boosting: 1,750 (6 months)	Pain at the injection site	[33, 42]
ChAd3	Group 1 (2.5 × 10^10^ vp) Group 2 (5 × 10^10^ vp)	EBOV; SUDV	GMC: Group 1: 51 μg/ml (Day 28) Group 2: 44.9 μg/ml (Day 28)	Fatigue	[43]
ChAd3+MVA	Priming: ChAd3 Group 1 (1 × 10^10^ vp) Group 2 (2.5 × 10^10^ vp) Group 3 (5 × 10^10^ vp) Group 4 (1 × 10^11^ vp) Boosting: MVA 2 × 10^8^ vp	EBOV; SUDV; MARV; TAFV	GMT: Priming Group 1: 295.0 (Day 28) Group 2: 204.6 (Day 28) Group 3: 555.8 (Day 28) Group 4: 1,493.6 (Day 28) Boosting: 9,279.6 (Day 28)	Pain at the injection site	[39]
rVSV	Sites 1&2: Group 1 (3 × 10^6^ PFU) Group 2 (2 × 10^7^ PFU) Site 3: Group 1 (3 × 10^5^ PFU) Group 2 (3 × 10^6^ PFU) Site 4: Group 1 (1 × 10^7^ PFU) Group 2 (5 × 10^7^ PFU)	EBOV	GMT: Site 1: Group 1: 1392.9 (Day 28) Group 2: 1969.8 (Day 28) Site 2: Group 1: 1492.9 (Day 28) Group 2: (-) (Day 28) Site 3: Group 1: 1055.6 (Day 28) Group 2: 2570.9 (Day 28) Site 4: Group 1: 1064.2 (Day 28) Group 2: 1780.1 (Day 28)	Pain at the injection site	[24]
rVSV	Group 1 (3 × 10^5^ PFU)	EBOV	GMT: Group 1: 344.5 (Day 28)	Pain at the injection site	[44]
rVSV	Group 1 (3 × 10^6^ PFU) Group 2 (2 × 10^7^ PFU)	EBOV	GMT: Group 1: 1,300 (Day 28) Group 2: 4,079 (Day 28)	Pain at the injection site	[25]
rVSV	Group (2 × 10^7^ PFU)	EBOV	-	Pain at the injection site	[26]
DNA	Group 1 (2.0 mg) Group 2 (4.0 mg) Group 3 (8.0 mg)	EBOV; SUDV	-	Local reactions	[15]
DNA	Priming: Group (4.0 mg) Boosting: Group (4.0 mg)	EBOV; SUDV; MARV	GMT: Group: 31.8 (Day 28)	Pain at the injection site	[45]
DNA	Group (4.0 mg)	EBOV; SUDV; MARV	GMT: Group: 31.0 (Day 28)	Pain at the injection site	[46]
VSV+Ad5	Group 1: Priming VSV 1.25 × 10^7^ PFU Boosting Ad 1.25 × 10^11^ vp Group 2: Priming VSV 2.5 × 10^7^ PFU Boosting Ad 2.5 × 10^11^ vp	EBOV	Group 1: 33.51 (Day 21) 343.1 (Day 28) 2,540 (Day 42) Group 2: 55.49 (Day 21) 1,230 (Day 28) 3,277 (Day 42)	Pain at the injection site	[40]

Another way to solve the problem of pre-existing immunity to a vaccine vector is to use recombinant vector serotypes with rare pre-existing immunity in the human population [31]; e.g., human adenovirus serotype 26 or adenovirus chimpanzee serotype 3 (Ad3).

The Ad3-based vaccine passed phase 1 clinical trials and progressed to phases 2 and 3. Ad3-based vaccine vectors carry the *GP *gene of the Mayinga-Zaire 1976 Ebola virus or the *GP *gene of the Gulu-Sudan Ebola virus. The results of phase 1 clinical trials conducted in the United States [32] demonstrated that the vaccine induced a high level of GP-specific antibodies (titer of 1 : 2,037) and a T-cell response, which were associated with protectivity in a NHP model. However, clinical trials in England [33] reported a low titer (1 : 469) of GP-specific antibodies (mean values did not reach levels protective for primates); the T-cell response had a maximum at day 14 of the study and decreased by day 28.

One of the problems of the developed vaccines for EVD is the reduction in the protective immune response a few months after immunization. This problem can be solved by using heterologous prime-boost vaccination (*Figure 1*). This vaccination strategy against Ebola was recommended by the WHO as the most promising one [34]. It should also be noted that recombinant viral vectors have certain disadvantages (pre-existing immunity to an Ad5-based vaccine vector [35], incorrect processing of target antigens when using a MVA-based vaccine vector [36], and lack of data on the duration of the protective immune response for VSV-based vaccine vectors [37]) that can be eliminated by using heterologous vaccination (*Figure 1*).

A series of preclinical trials in primates [38] demonstrated that homologous vaccination with an Ad3 (Ad3 + Ad3)-based vector results in 100% short-term protection (5 weeks); but with this vaccination regimen, protection decreased to 33% in 8 months. For heterologous (Ad3 + MVA) vaccination, protection was 100% 8 months after boosting.

A heterologous vaccine based on Ad3 and recombinant Modified Vaccinia Ankara (MVA) virus passed phase 1 clinical trials. Ad3-based vaccine vectors carry the *GP *gene of the Mayinga-Zaire 1976 Ebola virus or the Gulu-Sudan Ebola virus; MVA vectors (multivalent MVA-BN-filo) carry the *GP *genes of EBOV, SUDV, and MARV and the *NP *gene of TAFV. The use of heterologous vaccination enabled a many-fold amplification of both the humoral and cellular immune responses [39]. Furthermore, the use of a vaccine based on an Ad3 and MVA combination preserved high titers (1 : 1,750) of GP-specific antibodies 6 months after boosting.

In Russia, a heterologous combined vectored EVD vaccine for prime-boost vaccination was developed in accordance with the WHO recommendations. It was based on two recombinant viral vectors expressing the Ebola virus glycoprotein: a recombinant vesicular stomatitis virus (VSV-GP) and a recombinant human adenovirus serotype 5 (Ad-GP) [40].

A series of preclinical trials in primates demonstrated that immunization with this vaccine provides 100% protection from infection to animals both 3 weeks after immunization and 5 months after immunization.

Clinical trials of safety and immunogenicity demonstrated that the vaccine provides high safety and immunogenicity levels to healthy volunteers.

No serious adverse events (AEs) occurred during the vaccine safety study. All AEs were mild or moderate, developed within the first 2 days after vaccination, and resolved within the next 3 days. The most common AEs were pain at the injection site, headache, and weakness/fatigue. These AEs are typical of most recombinant viral vectored vaccines.

The vaccine efficacy was assessed using various parameters of the humoral and cellular immune responses: The seroconversion level was 100%. The mean titer of ZEBOV-GP-specific IgGs on day 42 of the study was 1 : 3,277 in a group receiving a full dose of the vaccine. Importantly, immunization with VSV-GP alone, at the same dose, induced antibodies in a titer of 1 : 538 to day 42, which was significantly lower than the titers obtained with heterologous vaccination. On day 28, a virus neutralization assay detected virus neutralizing antibodies with a mean titer of 1 : 20 in 93.1% of volunteers receiving a full dose of the vaccine. The cellular immune response was assessed by IFN-gamma production in peripheral blood mononuclear cells after antigen challenge: a response was detected in 100% of the volunteers on day 42 of the study.

Despite the published data on the negative effect of pre-existing immunity to adenoviruses, there was no significant correlation between the level of Ad5 neutralizing antibodies and the level of a GP-specific humoral and cellular response in the case of immunization of healthy volunteers with the VSV- and Ad-based vaccine. This indicates that the use of heterologous vaccination neutralizes the negative effect of pre-existing immunity to human adenovirus serotype 5-based vaccine vectors.

Based on the findings of preclinical and clinical trials demonstrating its high vaccine efficacy and safety, the EVD vaccine developed and produced at the Gamaleya Research Center for Epidemiology and Microbiology was licensed in the Russian Federation in 2015.

## CONCLUSION

EVD poses a serious threat to global security. Since 1976 when the Ebola virus was first detected, more than 20 outbreaks have been recorded. They have mainly occurred in the rural areas of East and Central Africa. But in 2014, the outbreak that began in three countries in West Africa changed the situation. These were the first cases when the virus was detected in urban centers, and the virus could spread outside of Africa to Europe and North America.

The spread of the Ebola virus outside of Africa during the 2014–2016 EVD epidemic and the high mortality rate were a solid reason for the active development of effective preventive and therapeutic remedies. To date, various clinical trials in Africa, Europe, the U.S., and Russia have shown the good safety and immunogenicity profiles of several EVD vaccines. Eight vaccines are now under different phases of clinical trial. Two vaccines (“GamEvac” and “GamEvac-Combi”) developed and produced at the Gamaleya Research Center for Epidemiology and Microbiology are currently the only licensed vaccines for Ebola fever: The “GamEvac-Combi” vaccine is a heterologous VSV- and Ad5-vectored vaccine, and the “GamEvac” vaccine is a homologous Ad5-vectored vaccine.

In conclusion, it should be noted that despite the high price already paid, mankind has learned an important lesson: It has become obvious that a timely drive against global threats to public health is possible only if the efforts of political leaders, WHO experts, and key pharmaceutical players are consolidated. The combined work of experts from different fields enabled the fast introduction of novel advanced vaccines into practical medicine.

Obviously, the gained experience will be used in the future for the timely development of vaccines for other dangerous viral infections, the preventive measures for which are absent at the moment (severe acute respiratory syndrome, Middle East respiratory syndrome caused by coronavirus, Zika virus disease, etc.).

## Abbreviations

Ad3, ChAd3: recombinant replication-defective chimpanzee adenovirus type 3
Ad5: recombinant replication-defective human adenovirus type 5
Ad26: recombinant replication-defective human adenovirus type 26
BDBV: Bundibugyo Ebolavirus
GMT: geometric mean titer
GMC: geometric mean concentration
GP: glycoprotein
MARV: Marburg virus
MVA: recombinant Modified Vaccinia Virus Ankara
NP: nucleoprotein
RESTV: Reston Ebolavirus
SUDV: Sudan Ebolavirus
TAFV: Tai Forest Ebolavirus
VSV: live-attenuated recombinant vesicular stomatitis virus
ZEBOV: Zaire Ebolavirus
EVD: Ebola virus disease
PFU: plaque-forming unit
NtAb: virus-neutralizing antibody
VP: viral particle
IFN: gamma – interferon gamma
AE: adverse event
